# The Dutch Sensory Perception Quotient-Short in adults with and without autism

**DOI:** 10.1177/1362361320942085

**Published:** 2020-07-28

**Authors:** Ricarda F Weiland, Tinca JC Polderman, Rosa A Hoekstra, Dirk JA Smit, Sander Begeer

**Affiliations:** 1 Vrije Universiteit Amsterdam, The Netherlands; 2 King’s College London, UK; 3 Academic Medical Center, Amsterdam, The Netherlands

**Keywords:** autism, perception, questionnaire, sensory sensitivity, SPQ

## Abstract

Sensory symptoms were recently added to the diagnostic criteria of autism spectrum disorder and may be a mechanism underlying the broad phenotype of autism spectrum disorder. To measure sensory symptoms based on perceptual rather than affective, regulative, or attention components, the Sensory Perception Quotient (SPQ) measuring five modalities of sensory sensitivity has been developed. In this study, the Dutch translation of the abridged SPQ-Short was investigated in a large sample of adults with (*n* = 657) and without autism spectrum disorder (*n* = 585). Its hypothesized factor structure, combining modality specific and one modality-independent factor, was assessed in a hierarchical model. Results show that modality-specific subscales are indeed present in the short version. Furthermore, its reliability is high and comparable to the original English version. The autism spectrum disorder group reported higher sensory sensitivities than the comparison group, and women with autism spectrum disorder reported higher sensitivities compared with men with autism spectrum disorder. The SPQ-Short correlates with all Autism Quotient (AQ)-Short subscales, except for the “imagination” subscale. The SPQ-Short seems suitable to further explore the relationship between basic sensory sensitivities in autism spectrum disorder and their related symptoms such as over- and under-responsivity to sensory stimulation.

**Lay Abstract:**

Individuals on the autism spectrum often experience heightened or reduced sensory sensitivities. This feature was recently added to the diagnostic manual for autism (*Diagnostic and Statistical Manual of Mental Disorders*, 5th ed. (DSM-5)). To measure sensory sensitivities, the Sensory Perception Quotient (SPQ) has been developed. In this study, we tested whether a Dutch translation of the abridged SPQ-Short yields similar results as the original English version. We also tested whether this questionnaire can measure modality specific sensitivities. To this end, 657 adults with autism spectrum disorder and 585 adults without an autism spectrum disorder diagnosis filled out the Dutch SPQ-Short. The Dutch questionnaire data were very similar to the original English version: adults with autism spectrum disorder were more sensitive compared with adults without autism spectrum disorder. Women with autism spectrum disorder are more sensitive compared with men with autism spectrum disorder. Gender did not have an effect in the group without autism spectrum disorder. Individuals reporting higher sensory sensitivities also reported more autistic traits (such as lower social interests, or increased fascination for patterns). Finally, we found that the Dutch SPQ-Short is suited to measure modality-specific sensitivities. We conclude that the Dutch translation is a viable tool to measure sensory sensitivities in adults with and without autism spectrum disorder and can be used to further our understanding of differences in perception in people with or without autism spectrum disorder.

Autism spectrum disorder (ASD) is defined by both social (deficits in social-emotional reciprocity, nonverbal communication, and relationships) and non-social (stereotyped motor movements, insistence on sameness, and fixated intense interests) symptoms. The most recent edition of the *Diagnostic and Statistical Manual of Mental Disorders* (DSM; American Psychiatric Association, 2013) and the new draft edition of the ICD (World Health Organization, n.d.) added sensory symptoms to the diagnostic criteria of ASD. In the *Diagnostic and Statistical Manual of Mental Disorders* (5th ed.; DSM-5; American Psychiatric Association, 2013), those sensory symptoms are defined as “hyper- or hyporeactivity to sensory input or unusual interest in sensory aspects of the environment” (American Psychiatric Association, 2013, p. 50). While the DSM defines hyper- or hyporeactivity as behaviors that are observable by others for the clinical practice (e.g. “apparent indifference to pain/temperature”), basic research usually focuses more on measurable standardized concepts such as responses in stimulus detection or discrimination tasks. In this context, sensory symptoms are usually referred to as hyper- or hyposensitivity (Schauder & Bennetto, 2016).

While enhanced sensory sensitivity has been described as a symptom of ASD (e.g. weak central coherence, Frith, 2003; enhanced perceptual functioning, Mottron et al., 2006), more recent studies suggest that atypical sensory sensitivity may be a factor contributing to higher order processes (e.g. social cognition) and could explain other ASD symptoms (e.g. Baum et al., 2015; Mottron et al., 2006; Pellicano & Burr, 2012; Rubenstein & Merzenich, 2003). Sensory symptoms in this sense are defined as perceptual differences, excluding other cognitive, affective, and behavioral aspects as much as possible (Schauder & Bennetto, 2016). Importantly, it is suggested that enhanced sensory sensitivities have a central origin, meaning that differences are expected to be in the central nervous system, not in the periphery of individual organs, such as eyes or ears (Ward, 2019; however, see Orefice et al., 2016, who have found evidence for peripheral causes for tactile sensitivity in mouse models). This would lead to modality-independent enhanced sensory sensitivity which has been implied by multiple questionnaire studies in individuals with ASD (Kuiper et al., 2019a; Tavassoli et al., 2014) and in typically developing individuals (Robertson & Simmons, 2013). Yet, on the contrary, more clinically oriented studies do report modality-specific symptoms (Landon et al., 2016; Robertson & David, 2015) and most questionnaires investigating sensory sensitivities include modality-specific subscales (Brown et al., 1997; Robertson & Simmons, 2013; Schoen et al., 2002; Tavassoli et al., 2014).

One issue with most sensory sensitivity questionnaires (for an overview, see Burns et al., 2017) is that they focus on observable behavior and do not differentiate between atypical sensory sensitivity, reactivity, or other related processes such as attention or perceptual load (Schauder & Bennetto, 2016). For example, the most widely used AASP includes items covering aspects such as social and communication abilities (e.g. “I do not get jokes as quickly as others”) or attention (e.g. “I miss the street, building, or room signs when trying to go somewhere new”). It therefore measures primarily cognitive and affective responses toward stimuli, and not sensory sensitivities. Although highly useful in clinical practice, its use in basic research aiming to investigate sensory processing differences as an underlying perceptual mechanism of ASD is limited.

The Sensory Perception Quotient (SPQ) was developed to address this gap and specifically measures sensory sensitivity independent of reactive behaviors or complex cognitive processes (Tavassoli et al., 2014). Items assess whether a person is able to detect or distinguish stimuli in the environment (e.g. “I can see dust particles in the air in most environments,” or “I would notice if someone added 5 drops of lemon juice to my cup of water”) and are rated on a 4-point Likert-type scale. Previous research has found that individuals with ASD show overall higher sensory sensitivities compared with neuro-typical individuals (Greenberg et al., 2018; Tavassoli et al., 2014). SPQ scores correlated with general autistic traits both overall and within groups, indicating that sensory sensitivity and autistic traits are associated even in individuals without an ASD diagnosis. The SPQ has five theoretically driven subscales, one for each of the modalities vision, hearing, touch, smell, and taste which are scored independently. Items for the SPQ-Short were chosen by a principal component analysis (see Tavassoli et al., 2014, for details). Although in the long version, half of the items are assessing hypersensitivity/hyposensitivity, in the SPQ-Short most items are assessing hypersensitivity.

The SPQ is the first questionnaire showing that sensory sensitivity differences exist on a perceptual level, making it a promising tool for investigating the non-affective part of sensory sensitivities. However, so far only an English version is available. This study reports on the Dutch translation of the SPQ-Short, in a large cohort of adults with and without ASD. We extend previous psychometric evaluation of the instrument by examining the factor structure of the SPQ-Short, as well as its reliability and conceptual validity by analyzing group differences and the correlation between the SPQ-Short and distinct autism domains, assessed by the Autism Spectrum Quotient-Short (AQ-Short) (Hoekstra et al., 2011).

## Methods

The protocol of this study was approved by the ethics committee of the VU University Medical Center (approval number 2013/45) and all participants provided written informed consent. Data and scripts from this study are available upon request.

### Participants

Participants included adults with a formal ASD diagnosis (*n* = 657) as well as a neuro-typical adult comparison group (*n* = 585). All ASD participants reported having received a formal diagnosis by a qualified clinician unaffiliated to this study of either ASD according to the DSM-5, or autistic disorder, Asperger syndrome, childhood disintegrative disorder, or pervasive developmental disorder not otherwise specified (PDD-NOS) according to the *Diagnostic and Statistical Manual of Mental Disorders* (4th ed.; DSM-IV; American Psychiatric Association, 1994). Participants were recruited through the Netherlands Autism Register (NAR, www.nederlandsautismeregister.nl/english/), a research volunteer register for individuals with ASD and neurotypical participants who are invited to participate in online surveys at yearly intervals. Participants for this study completed an online version of the SPQ-Short as well as the AQ-Short (Hoekstra et al., 2011), and reported various demographical information, such as age and gender. Table 1 gives descriptive data for all participants who were included in the current study.

**Table 1.** Demographical data of participants.

		ASD group	Control group	Group difference
N		657	585	–
Proportion female^a^		51.9%	74.4%	*p* < 0.001
Age in years (*SD*)		43.2 (13.5)	38.4 (14.9)	*p* < 0.001 Cohen’s *d* *=* 0.34
AQ-Short score (*SD*)		83.2 (11.5)	51.7 (10.6)	*p* < 0.001 Cohen’s d = 2.84
Educational Level	High	295 (44.9%)	313 (53.5%)	Chi^2^ = 74.71 *p* < 0.001
	Middle	257 (39.1%)	78 (13.3%)	
	Low	16 (2.4%)	4 (0.6%)	
Ethnicity	Only Dutch	597 (90.8%)	501 (85.6%)	Chi^2^ = 33.69 *p* < 0.001
	Partly Dutch, or Other^b^	27 (4.1%)	80 (13.7%)	

ASD: autism spectrum disorder; AQ: autism quotient.

a In the control group, *n* = 4 (0.6%) of participants indicated their gender as being “other.” ^b^ Other ethnicities in this sample include mainly Surinamese, Indonesian, and Indian.

Age and gender differed significantly between the ASD and comparison group with the ASD group being older and having more male participants. Exploratory analyses showed that age did not correlate significantly with the SPQ-Short score (*r* = 0.06, *p* = 0.118) and gender did not have a significant effect (*t* = −1.46, *p* = 0.146).

### Measures

#### Sensory Perception Quotient-Short

The SPQ-Short (Tavassoli et al., 2014) is a shortened version of the SPQ consisting of 35 items assessing sensory sensitivity in five different sensory modalities (vision, hearing, taste, touch, smell). The items were derived from a pool of 92 items following an item reduction procedure using Principal Component Analysis (see Tavassoli et al., 2014, for details). Items contain statements about detecting a certain stimulus (e.g. “I would feel if a single hair touched the back of my hand.”) which participants rate on a 4-level Likert-type scale (“definitely agree”; “slightly agree”; “slightly disagree”; and “definitely disagree”). Items are scored between 0 and 3, with 0 indicating high sensitivity and 3 indicating low sensitivity. So far, only the total score (sum of all item scores) has been used since the factor structure of the SPQ-Short has not previously been examined.

For the present study, the SPQ-Short was translated to Dutch using the backward translation procedure (see items Supplemental Table S1). One item (35) has unfortunately been erroneously translated as a positive item, whereas the original English item is negative. For the analyses, it was coded as a positive item.

Participants with more than three missing items (>10% of the items) were excluded from further analyses (*n* = 20). Of the remaining participants, *n* = 54 had one missing item, and *n* = 7 had two missing items (overall 0.1% of items were missing). Those missing values were imputed using two-way imputation (van Ginkel et al., 2010).

#### Autism Quotient-Short

The AQ-Short (Hoekstra et al., 2011) consists of 28 items categorized into two higher-order factors (“social behavior” and “fascination for numbers/patterns”). The higher order factor “social behavioral difficulties” is further divided into the subfactors “social skills,” “routine,” “attention switching,” and “imagination.” Each item is comprised of a statement (e.g. “I find social situations easy”) on which the participant agrees to on a 4-level Likert-type scale (‘definitely agree’; ‘slightly agree’; ‘slightly disagree’; and “definitely disagree”). Items are scored between 1 and 4, with higher scores indicating higher autistic traits. The total score is the sum of all items scores, subscale scores are the sum of all item scores belonging to that subscale. Cronbach’s alpha values for the complete Dutch AQ-Short is acceptable (between 0.77 and 0.79) as are values for subscales (between 0.70 and 0.80).

### Statistical approach

All analyses were done in RStudio version 1.1.463, using the package lavaan for the confirmatory factor analysis (Rosseel, 2012; RStudio Team, 2015). To assess the factor structure of the SPQ-Short, a confirmatory factor analysis approach was used to investigate a hierarchical model where items first loaded on their respective sensory modality (vision, hearing, touch, smell, and taste; see Tavassoli et al., 2014), which in turn load onto a single factor (“sensory sensitivity”). The confirmatory factor analysis was performed on the polychoric correlation matrix of items, using the WLSVM estimator (Holgado–Tello et al., 2010). To determine model fit, the following indices were examined: comparative fit index (CFI), root mean square errors of approximation (RMSEA), and standardized root mean square residuals (SRMR). Chi-square test statistics and *p* values are also be reported when available. The goal was to achieve at least an adequate fit with a CFI > 0.9, and RMSEA and SRMR < 0.08 (Hooper et al., 2008; Hu & Bentler, 1999; Schermelleh-Engel et al., 2003). In addition, a group-independent model (model 2) was assessed by equalizing path loadings between both groups to assess whether the factor structure differed between groups. Both models were compared using an analysis of variance (ANOVA).

The reliability of the SPQ-Short was assessed with the mean of 1000 Spearman-Brown split-half correlations and by calculating Cronbach’s alpha. Furthermore, group differences between ASD and comparison group were investigated. Correlations between the SPQ-Short score and AQ-Short total score, and each AQ-Short subscale score, were calculated using Kendall’s tau since it provides more reliable results than Spearman’s correlation for non-normally distributed data, as well as better interpretable confidence intervals (Newson, 2002). Confidence intervals (95%) were calculated by bootstrapping 1000 times.

## Results

### Confirmatory factor analyses

**Figure 1.**
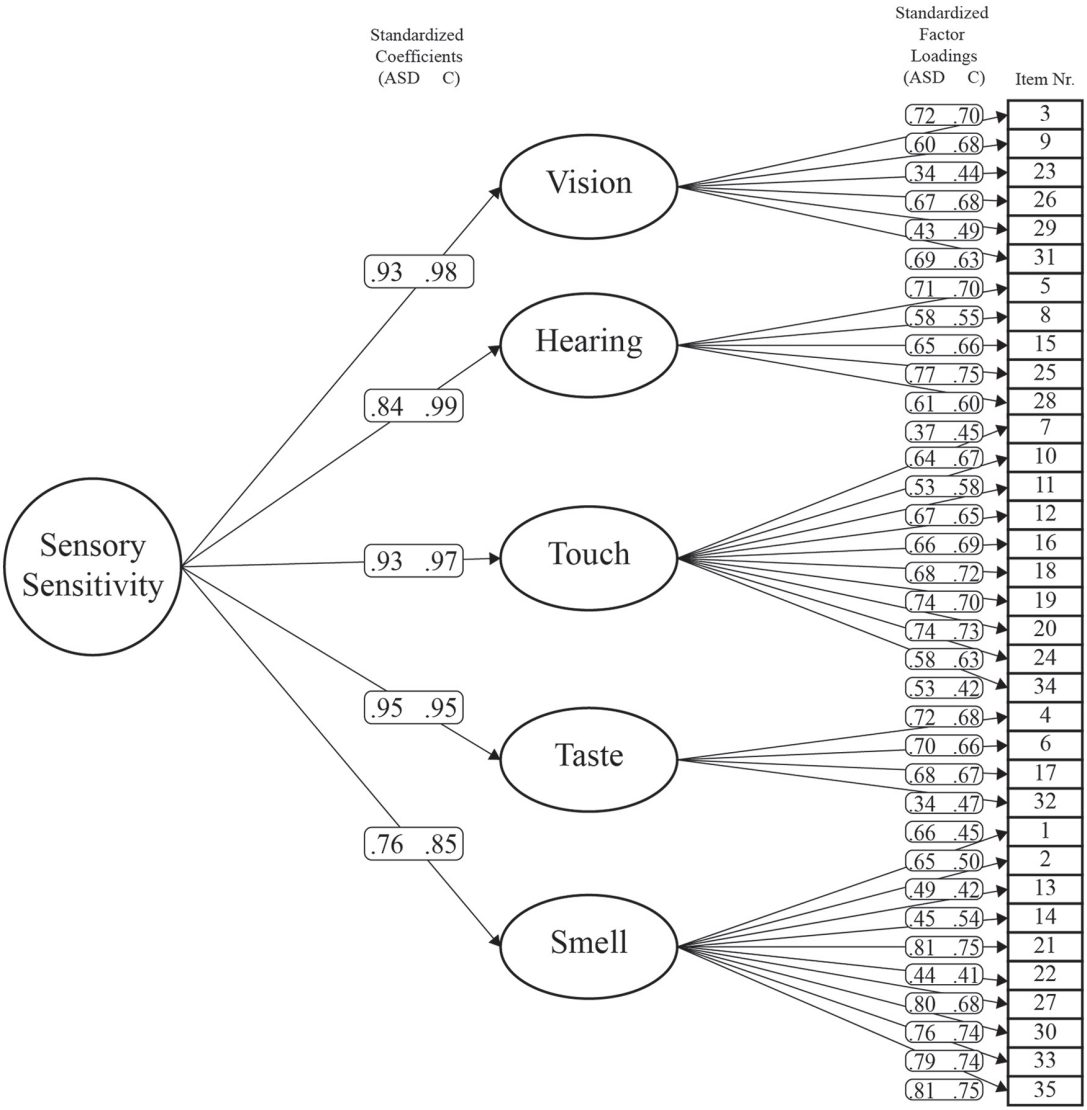
Hierarchical factor model of the SPQ-Short. Depicted are standardized factor loadings per group (ASD left, control right). Items (squares) first load onto their respective modality specific factor. All modality specific factors load in turn onto the higher-order modality-independent factor.

The hierarchical model was assessed per group (model 1). Model fit indices indicated an excellent fit (CFI = 0.975, RMSEA = 0.064, SRMR = 0.062) and met the pre-determined cutoff criteria (see Table 2). Comparing model 1 with model 2 (the one not accounting for group differences) yielded a significant result, indicating that groups do indeed differ (*p* < 0.001). Inspection of model 1 showed that path loadings were usually lower in the ASD group (see Figure 1).

**Table 2.** Goodness-of-fit indicators of hierarchical models.

	Number of parameters	Chi^2^	df	*p* value	CFI	RMSEA	RMSEA CI-lower	RMSEA CI-upper	SRMR
Model 1	290	3907.52	1110	<0.001	0.975	0.064	0.062	0.066	0.062
Model 2^a^	250	4794.58	1150	<0.001	0.968	0.071	0.069	0.074	0.070

a In model 2, path loadings were kept consistent across both groups.

CFI: comparative fit index, RMSEA: root mean square error of approximation, CI: 95% confidence interval, SRMR: standardized root mean square residual.

### Reliability

The mean of 1000 estimated split-half correlations was high (Spearman-Brown = 0.87), as well as Cronbach’s alpha (α = 0.93).

### Group differences

**Figure 2.**
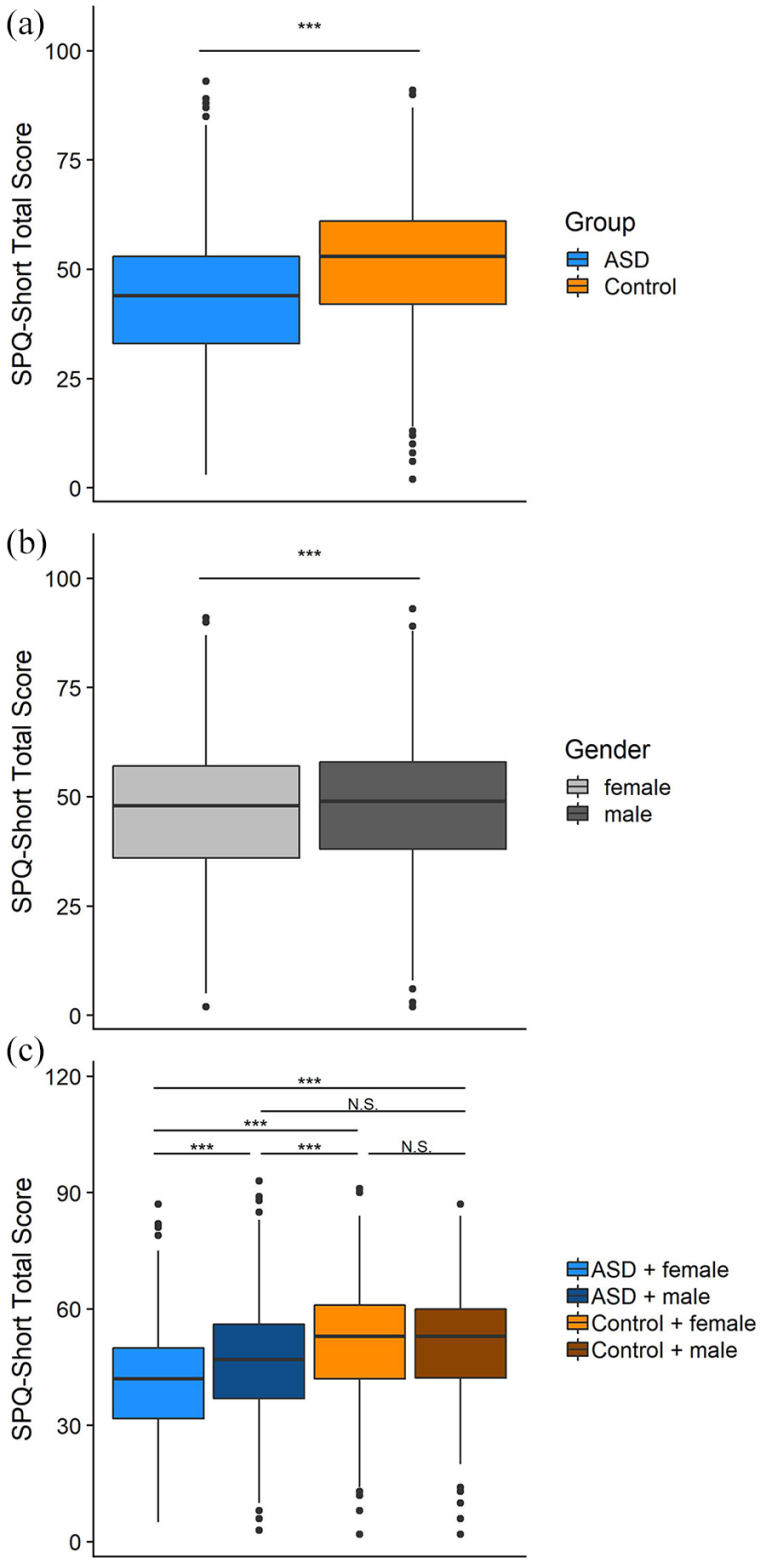
SPQ-Short scores distributions. Figure 2(a) shows distribution by group and (b) shows the distribution by gender; (c) shows distributions per gender and group. Horizontal black lines indicate medians, upper and lower bounds correspond to 75th and 25th percentiles, respectively. Whiskers extend until 1.5 the interquartile range, black dots indicate outliers beyond this.

Group differences were assessed on the total score of the SPQ-Short and the modality-specific subscales. Since the data were not normally distributed (Shapiro–Wilk normality test: *W* = 1.00, *p* = 0.001) but did have variance homogeneity (Levene test: *F* = 0.27, *p* = 0.600), a Wilcoxon rank sum test was performed. This revealed a significant group difference (*W* = 136287, *p* < 0.001) with a small effect size (Cohen’s *d* = −0.45, see Figure 2(a) and Table 3). The ASD group scored lower than the comparison group, indicating higher sensory sensitivity in the ASD group. This pattern was consistent for all subscales, except for smell where no significant group effect was found (see Table 3).

**Table 3.** SPQ-Short total scores.

SPQ-Short total score	ASD			Control		
	Overall	Female	Male	Overall^a^	Female	Male
Mean	43.67	40.70	46.87	50.69	51.04	49.64
*SD*	15.34	14.33	15.77	15.79	15.62	16.28

ASD: autism spectrum disorder; SPQ: Sensory Perception Quotient.

a Four control participants indicating their gender as being “other” are included in the overall score.

To test for potential gender effects, a two-way ANOVA was performed with the main effects group (with versus without ASD) and gender (female versus male, see Table 3). For these analyses, four participants without ASD who indicated their gender as “other,” were excluded. Results showed that both main effects were significant, with the ASD group, and females reporting more sensitivity (Group: *F*(1, 1234) = 86.15, *p* < 0.001; Gender: *F*(1, 1234) = 26.26, *p* < 0.001) as was the interaction term (*F*(1, 1234) = 15.81, *p* < 0.001). Post hoc tests (Tukey’s) showed that the effect of gender was only significant in the ASD group (*p* < 0.001) with women reporting higher sensory sensitivities compared with men, but no gender effect in the comparison group (*p* = 0.778, see Figure 2(c)).

### Correlation between SPQ-Short score and AQ-Short scores

**Figure 3.**
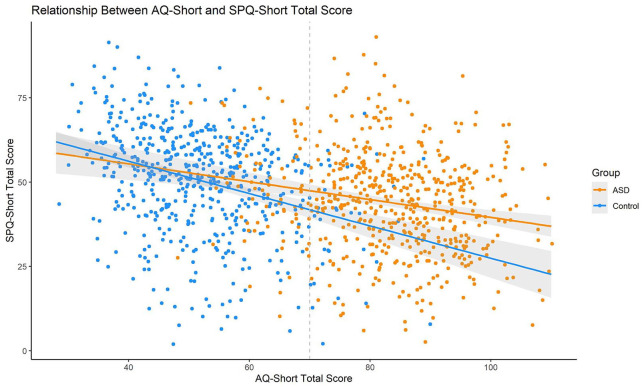
Relationship between AQ-Short and SPQ-Short total scores. Colored lines indicate linear regressions, the gray area around them are 95% confidence intervals. The vertical dotted gray line indicates the AQ-Short cutoff of 70, as suggested by Hoekstra et al. (2011).

The τ correlation coefficient between SPQ-Short and AQ-Short total scores was rather small in both the ASD group (τ = −0.13, *p* < 0.001) and the comparison group (τ = −0.23, *p* < 0.001), indicating that higher sensitivities are positively associated with higher autistic traits (see Figure 3).

Investigation of the AQ-Short subscale scores revealed that “imagination” was the only subscale that did not correlate significantly in either group (with ASD: τ = 0.04, *p* = 0.119; without ASD: τ = −0.005, *p* = 0.873).

All other AQ-Short subscales correlated significantly with the SPQ-Short total score both in the ASD group (τ between −0.07 and −0.21, all *p*s < 0.008), and the comparison group (τ between −0.11 and −0.23, all *p*s < 0.001, see Table 4).

**Table 4.** Correlations between SPQ-Short total score and AQ-Short subscale scores.

AQ-Short subscale	ASD		Control	
	Correlation coefficient	*p* value	Correlation coefficient	*p* value
Numbers & Patterns	τ = −0.21 (−0.27, −.16)	<.001	τ = −.23 (−.29, −.18)	<.001
Social Behavior	τ = −0.07 (−0.13, −0.02)	0.003	τ = −0.17 (−0.22, −0.12)	<0.001
Social Skills	τ = −0.09 (−0.14, −0.03)	0.001	τ = −0.23 (−0.28, −0.18)	<0.001
Routine	τ = −0.14 (−0.19, −0.09)	<0.001	τ = −0.12 (−0.17, −0.06)	<0.001
Attention Switching	τ = −0.11 (−0.16, −0.06)	<0.001	τ = −0.16 (−0.21, −0.11)	<0.001
Imagination	τ = 0.04 (−0.11, 0.10)	0.119	τ = 0.0001 (−0.06, 0.06)	0.996

SPQ: Sensory Perception Quotient; AQ: autism quotient; ASD: autism spectrum disorder.

In brackets are 95% confidence intervals of Kendall’s tau based on bootstrapping 1000 times.

## Discussion

This study investigated sensory sensitivities in a large adult sample of adults with and without ASD, using the Dutch version of the SPQ-Short. Adults with ASD generally reported higher sensory sensitivity than adults without ASD, replicating previous findings (Greenberg et al., 2018; Tavassoli et al., 2014), and females with ASD reported higher sensory sensitivity compared with males with ASD. The SPQ-Short was correlated with autistic symptoms as measured with the AQ-Short, except for the AQ-Short subscale “imagination.” The five-factor structure fit well to the data, albeit that the structure differed significantly between ASD cases and control, largely the result of lower path loadings in the ASD group.

Although our finding of higher sensory sensitivity in individuals with ASD is in line with previous research and perceptual theories of ASD (Greenberg et al., 2018; Tavassoli et al., 2014), decreased (or hypo-) sensitivities which have been reported in clinical descriptions, and in the DSM-5 (American Psychiatric Association, 2013) are missing in this study. We consider two possible explanations for this: First, the SPQ-Short comprises questions mainly investigating hypersensitivity (*n* = 30 items, see Supplemental Table S1). Only five items are aimed at measuring hyposensitivity, which could have led to the increased finding of hypersensitivity. It should be noted however that in the full SPQ, half of the items are assessing hyposensitivity but were disproportionately excluded for the short version, possibly because it is easier to report on hypersensitivity than hyposensitivity (Schauder & Bennetto, 2016). Furthermore, even the items aimed at measuring hyposensitivity are simply reverse coded items for hypersensitivity. Second, previous research has shown that children and adolescents with ASD and average or above average intelligence show greater sensitivity compared with children with ASD and below average intelligence (Duerden et al., 2015; Mayes & Calhoun, 2011). It is quite possible that a similar relationship holds in adults with ASD. Since our sample consists solely of adults capable of filling in questionnaires, adults with lower cognitive abilities are under-represented.

Women with ASD showed higher SPQ-Short scores compared with men with ASD which is consistent with past research (Greenberg et al., 2018; Tavassoli et al., 2014). In adults without ASD, there was no significant effect of gender. This indicates that the gender difference is specific to individuals with ASD, and suggests that women with ASD are particularly hypersensitive. It is known that women with ASD are generally underdiagnosed (Begeer et al., 2013; Evans et al., 2019; Loomes et al., 2017), and in order to receive a diagnosis, women need to show stronger symptoms (Evans et al., 2019). Therefore, only women with higher sensory sensitivities might receive a diagnosis and are included in this study.

The SPQ-Short correlated significantly with the AQ-Short total score, within both groups. This shows that sensory sensitivity relates to autistic symptoms within individuals with ASD but also in the general population. Correlation coefficients were rather low but comparable to previous research using the SPQ-Short (Tavassoli et al., 2014). The small size of the correlation is a testament to the complexity of ASD: “sensory first theories” of ASD generally propose that initial small differences in perception add up once sensory information is further integrated (Baum et al., 2015). In line with this reasoning, studies using other sensitivity questionnaires that include higher order affective and attentional processes show comparatively larger correlation coefficients (e.g. Horder et al., 2014; Mayer, 2017; Robertson & Simmons, 2013). The SPQ-Short might be a valuable addition to tools used in clinical diagnoses, especially in women, since they tend to be highly sensitive and are often missed in diagnostic procedures.

Furthermore, all AQ-Short subscales showed a significant correlation with the SPQ-Short, except for the subscale “Imagination.” A similar lack of correlation between imagination and sensitivity has been found previously, using the AASP as a measure for sensitivity (Mayer, 2017). A possible explanation for this finding is that “Imagination” items measure processes that are less related to sensory stimulation and more related to internal processes (e.g. “Trying to imagine something, I find it easy to create a picture in my mind,” compared with e.g. “I am fascinated by dates”).

The factor structure of the SPQ-Short, with modality specific first-order factors, and a second-order factor for general sensory sensitivity, is in line with previous theories of abnormal perceptual functioning in ASD (Baum et al., 2015; Van de Cruys et al., 2013; Ward, 2019), as well as firsthand accounts of individuals with ASD (Landon et al., 2016; Robertson & David, 2015). The model accounting for group differences fitted better than the group-independent model, with generally lower path loadings in the ASD group (see Figure 3). However, we also observe that the group effects on the path loadings were small in absolute terms and reached significance due to the high power of the study.

As the SPQ-Short is a self-report measure, consciousness of and attention to small changes in their environment likely play a role in participant’s responses. Examples are items that could potentially measure being distracted easily by small environmental changes (e.g. “I would be able to visually detect the change in brightness of a light each time a dimmer control moved one notch.”), or by being able to focus on details in distracting environments (e.g. “If I look at a pile of blue sweaters in a shop that are meant to be identical, I would be able to see differences between them.”). This shows that even with a careful selection of items, a questionnaire may not capture purely objective sensory sensitivity.

Missing in this study is the investigation of the questionnaire’s concurrent validity. Tavassoli and colleagues (2014) used another sensitivity questionnaire (SensOR) to investigate this. An additional approach would be to compare SPQ-Short scores with measures from psychophysical detection tasks. Although this has not been investigated using the SPQ-Short, some studies have used other questionnaires to compare self-report with psychophysical measures in adults with ASD. Findings, however, remain mixed: while Karhson and Golob (2016), for example, did find a significant correlation between the self-reported and behavioral sensitivity in adults with ASD, other studies found little to no correlations (Jones et al., 2009; Kuiper et al., 2019b; Schulz & Stevenson, 2020; Williams et al., 2019). It is conceivable that results with the SPQ-Short would be clearer since its measurement is closer to sensory sensitivity than other questionnaires.

## Conclusions

This study presents the first investigation of the SPQ-Short in the Netherlands, using a well-powered sample of individuals with and without ASD. The Dutch SPQ-Short appears to be reliable, and as such a valuable instrument to measure perceptual sensory sensitivity in the Dutch population with and without ASD. The factor analysis of the SPQ-Short yielded inconclusive results, and needs further investigation. Our study replicates previous findings of SPQ measures in the United Kingdom, such as a heightened sensitivity in adults with ASD, a heightened sensitivity in females compared with males, and correlations between the SPQ-Short and AQ-Short. All AQ-Short subscales correlated with the SPQ-Short, except for the subscale “Imagination.”

## Supplemental Material

Suppl_S2 – Supplemental material for The Dutch Sensory Perception Quotient-Short in adults with and without autism Click here for additional data file. Supplemental material, Suppl_S2 for The Dutch Sensory Perception Quotient-Short in adults with and without autism by Ricarda F Weiland, Tinca JC Polderman, Rosa A Hoekstra, Dirk JA Smit and Sander Begeer in Autism

## References

[bibr1-1362361320942085] American Psychiatric Association. (1994). Diagnostic and statistical manual of mental disorders (4th ed.).

[bibr2-1362361320942085] American Psychiatric Association. (2013). Diagnostic and statistical manual of mental disorders. American Psychiatric Publishing.

[bibr3-1362361320942085] Baum S. H. Stevenson R. A. Wallace M. T. (2015). Behavioral, perceptual, and neural alterations in sensory and multisensory function in autism spectrum disorder. Progress in Neurobiology, 134, 140–160. 10.1016/j.pneurobio.2015.09.007 26455789 PMC4730891

[bibr4-1362361320942085] Begeer S. Mandell D. Wijnker-Holmes B. Venderbosch S. Rem D. Stekelenburg F. Koot H. M. (2013). Sex differences in the timing of identification among children and adults with autism spectrum disorders. Journal of Autism and Developmental Disorders, 43(5), 1151–1156. 10.1007/s10803-012-1656-z 23001766

[bibr5-1362361320942085] Brown C. Tollefson N. Cromwell R. Filion D. (1997). The adult sensory profile: Measuring patterns of sensory processing. American Journal of Occupational Therapy, 55, 75–82. 10.5014/ajot.55.1.75 11216370

[bibr6-1362361320942085] Burns C. O. Dixon D. R. Novack M. Granpeesheh D. (2017). A systematic review of assessments for sensory processing abnormalities in autism spectrum disorder. Review Journal of Autism and Developmental Disorders, 4(3), 209–224. 10.1007/s40489-017-0109-1

[bibr7-1362361320942085] Duerden E. G. Taylor M. J. Lee M. Mcgrath P. A. Davis K. D. Roberts S. W. (2015). Decreased sensitivity to thermal stimuli in adolescents with autism spectrum disorder: Relation to symptomatology and cognitive ability. The Journal of Pain, 16(5), 463–471. 10.1016/j.jpain.2015.02.001 25704841

[bibr8-1362361320942085] Evans S. C. Boan A. D. Bradley C. Carpenter L. A. (2019). Sex/gender differences in screening for autism spectrum disorder: Implications for evidence-based assessment. Journal of Clinical Child and Adolescent Psychology, 48, 840–854. 10.1080/15374416.2018.1437734 29601216 PMC6274603

[bibr9-1362361320942085] Frith U. (2003). Autism: Explaining the enigma. Blackwell Publishing.

[bibr10-1362361320942085] Greenberg D. M. Warrier V. Allison C. Baron-Cohen S. (2018). Testing the Empathizing-Systemizing theory of sex differences and the Extreme Male Brain theory of autism in half a million people. Proceedings of the National Academy of Sciences of the United States of America, 115(48), 12152–12157. 10.1073/pnas.1811032115 30420503 PMC6275492

[bibr11-1362361320942085] Hoekstra R. A. Vinkhuyzen A. A. Wheelwright S. Bartels M. Boomsma D. I. Baron-Cohen S. van der Sluis S. (2011). The construction and validation of an abridged version of the Autism-Spectrum Quotient (AQ-Short). Journal of Autism and Developmental Disorders, 41(5), 589–596. 10.1007/s10803-010-1073-0 20697795 PMC3076581

[bibr12-1362361320942085] Holgado–Tello F. P. Chacón–Moscoso S. Barbero–García I. Vila–Abad E. (2010). Polychoric versus Pearson correlations in exploratory and confirmatory factor analysis of ordinal variables. Quality and Quantity, 44(1), 153–166. 10.1007/s11135-008-9190-y

[bibr13-1362361320942085] Hooper D. Coughlan J. Mullen M. R. (2008). Structural equation modelling: Guidelines for determining model fit structural equation modelling. The Electronic Journal of Business Research Methods, 6(1), 53–60. www.ejbrm.com

[bibr14-1362361320942085] Horder J. Wilson C. E. Mendez M. A. Murphy D. G. (2014). Autistic traits and abnormal sensory experiences in adults. Journal of Autism and Developmental Disorders, 44(6), 1461–1469. 10.1007/s10803-013-2012-7 24305777 PMC4022987

[bibr15-1362361320942085] Hu L. T. Bentler P. M. (1999). Cutoff criteria for fit indexes in covariance structure analysis: Conventional criteria versus new alternatives. Structural Equation Modeling, 6(1), 1–55. 10.1080/10705519909540118

[bibr16-1362361320942085] Jones C. R. Happe F. Baird G. Simonoff E. Marsden A. J. Tregay J.. . .Charman T. (2009). Auditory discrimination and auditory sensory behaviours in autism spectrum disorders. Neuropsychologia, 47(13), 2850–2858. 10.1016/j.neuropsychologia.2009.06.015 19545576

[bibr17-1362361320942085] Karhson D. S. Golob E. J. (2016). Atypical sensory reactivity influences auditory attentional control in adults with autism spectrum disorders. Autism Research, 9, 1079–1092. 10.1002/aur.1593 26778164

[bibr18-1362361320942085] Kuiper M. W. M. Verhoeven E. W. M. Geurts H. M. (2019a). The Dutch Glasgow Sensory Questionnaire: Psychometric properties of an autism-specific sensory sensitivity measure. Autism, 23, 922–932. 10.1177/1362361318788065 30073851

[bibr19-1362361320942085] Kuiper M. W. M. Verhoeven E. W. M. Geurts H. M. (2019b). Stop making noise! Auditory sensitivity in adults with an autism spectrum disorder diagnosis: Physiological habituation and subjective detection thresholds. Journal of Autism and Developmental Disorders, 49, 2116–2128. 10.1007/s10803-019-03890-9 30680585 PMC6483953

[bibr20-1362361320942085] Landon J. Shepherd D. Lodhia V. (2016). A qualitative study of noise sensitivity in adults with autism spectrum disorder. Research in Autism Spectrum Disorders, 32, 43–52. 10.1016/j.rasd.2016.08.005

[bibr21-1362361320942085] Loomes R. Hull L. Mandy W. P. L. (2017). What is the male-to-female ratio in autism spectrum disorder? A systematic review and meta-analysis. Journal of the American Academy of Child and Adolescent Psychiatry, 56(6), 466–474. 10.1016/j.jaac.2017.03.013 28545751

[bibr22-1362361320942085] Mayer J. L. (2017). The relationship between autistic traits and atypical sensory functioning in neurotypical and ASD adults: A spectrum approach. Journal of Autism and Developmental Disorders, 47(2), 316–327. 10.1007/s10803-016-2948-5 27848052

[bibr23-1362361320942085] Mayes S. D. Calhoun S. L. (2011). Impact of IQ, age, SES, gender, and race on autistic symptoms. Research in Autism Spectrum Disorders, 5, 749–757. 10.1016/j.rasd.2010.09.002

[bibr24-1362361320942085] Mottron L. Dawson M. Soulières I. Hubert B. Burack J. (2006). Enhanced perceptual functioning in autism: An update, and eight principles of autistic perception. Journal of Autism and Developmental Disorders, 36(1), 27–43. 10.1007/s10803-005-0040-7 16453071

[bibr25-1362361320942085] Newson R. (2002). Parameters behind “nonparametric” statistics: Kendall’s tau, Somers’ D and median difference. The Stata Journal, 2(1), 45–64.

[bibr26-1362361320942085] Orefice L. L. Zimmerman A. L. Chirila A. M. Sleboda S. J. Head J. P. Ginty D. D. (2016). Peripheral mechanosensory neuron dysfunction underlies tactile and behavioral deficits in mouse models of ASDs. Cell, 166(2), 299–313. 10.1016/j.cell.2016.05.033 27293187 PMC5567792

[bibr27-1362361320942085] Pellicano E. Burr D. (2012). When the world becomes “too real”: A Bayesian explanation of autistic perception. Trends in Cognitive Sciences, 16(10), 504–510. 10.1016/j.tics.2012.08.009 22959875

[bibr28-1362361320942085] Robertson A. E. David R. S. R. (2015). The sensory experiences of adults with autism spectrum disorder: A qualitative analysis. Perception, 44(5), 569–586. 10.1068/p7833 26422904

[bibr29-1362361320942085] Robertson A. E. Simmons D. R. (2013). The relationship between sensory sensitivity and autistic traits in the general population. Journal of Autism and Developmental Disorders, 43(4), 775–784. 10.1007/s10803-012-1608-7 22832890

[bibr30-1362361320942085] Rosseel Y. (2012). {lavaan}: An {R} Package for structural equation modeling. Journal of Statistical Software, 48(2), 1–36. http://www.jstatsoft.org/v48/i02/

[bibr31-1362361320942085] RStudio Team. (2015). RStudio: Integrated development environment for R. http://www.rstudio.com/

[bibr32-1362361320942085] Rubenstein J. L. R. Merzenich M. M. (2003). Model of autism: Increased ratio of excitation/inhibition in key neural systems. Genes, Brain and Behavior, 2, 255–267. 10.1034/j.1601-183X.2003.00037.x PMC6748642 14606691

[bibr33-1362361320942085] Schauder K. B. Bennetto L. (2016). Toward an interdisciplinary understanding of sensory dysfunction in autism spectrum disorder: An integration of the neural and symptom literatures. Frontiers in Neuroscience, 10, Article 268. 10.3389/fnins.2016.00268 PMC4911400 27378838

[bibr34-1362361320942085] Schermelleh-Engel K. Moosbrugger H. Müller H. (2003). Evaluating the fit of structural equation models: Tests of significance and descriptive goodness-of-fit measures. MPR-Online, 8(2), 23–74.

[bibr35-1362361320942085] Schoen S. A. Miller L. J. Green K. E. (2002). Pilot study of the Sensory Over-Responsivity Scales : Assessment and inventory. American Journal of Occupational Therapy, 62(4), 393–406. 10.5014/ajot.62.4.393 18712002

[bibr36-1362361320942085] Schulz S. E. Stevenson R. A. (2020). Differentiating between sensory sensitivity and sensory reactivity in relation to restricted interests and repetitive behaviours. Autism, 24, 121–134. 10.1177/1362361319850402 31132855

[bibr37-1362361320942085] Tavassoli T. Hoekstra R. A. Baron-Cohen S. (2014). The Sensory Perception Quotient (SPQ): Development and validation of a new sensory questionnaire for adults with and without autism. Molecular Autism, 5, Article 29. 24791196 10.1186/2040-2392-5-29 PMC4005907

[bibr38-1362361320942085] Van de Cruys S. de-Wit L. Evers K. Boets B. Wagemans J. (2013). Weak priors versus overfitting of predictions in autism: Reply to Pellicano and Burr (TICS, 2012). i-Perception, 4(2), 95–97. 10.1068/i0580ic 23755353 PMC3677336

[bibr39-1362361320942085] van Ginkel J. R. Sijtsma K. Van Der Ark L. A. (2010). Incidence of missing item scores in personality measurement, and simple item-score imputation. Methodology, 6, 17–30. 10.1027/1614-2241/a000003

[bibr40-1362361320942085] Ward J. (2019). Individual differences in sensory sensitivity: A synthesizing framework and evidence from normal variation and developmental conditions. Cognitive Neuroscience, 10, 139–157. 10.1080/17588928.2018.1557131 30526338

[bibr41-1362361320942085] Williams Z. J. Failla M. D. Davis S. L. Heflin B. H. Okitondo C. D. Moore D. J. Cascio C. J. (2019). Thermal perceptual thresholds are typical in autism spectrum disorder but strongly related to intra-individual response variability. Scientific Reports, 9, Article 12595. 10.1038/s41598-019-49103-2 PMC6715703 31467358

[bibr42-1362361320942085] World Health Organization. (n.d.). ICD-11. https://icd.who.int/en/

