# Supplementary material for: The Dutch Sensory Perception Quotient-Short in adults with and without autism
Source: Autism. 2020 Jul 28;24(8):2071–80. doi: 10.1177/1362361320942085 (PMC7543020; doi:10.1177/1362361320942085)
Supplement: Suppl_S2 – Supplemental material for The Dutch Sensory Perception Quotient-Short in adults with and without autism [file Suppl_S2.pdf]

S2.

SPQ-Short Group Differences while controlling for Gender and Age.

Since the ASD and comparison group differed significantly both on gender and age (see Table 1), we repeated group difference analyses on gender and age-matched subgroups of the sample using the R-package MatchIt (Ho, Imai, King, & Stuart, 2011). Trying to match groups on both gender and age at the same time yielded unsatisfying results since matched subgroups still differed significantly on both age and gender. Therefore, matching was done for both variables apart.

Gender Matched Samples.

Matching on gender using the exact method yielded two groups with the same gender proportions (n = 341 women, n = 146 men). Repeating the Wilcoxon Rank Sum test on this subsample yielded similar results: the ASD group scored lower on the SPQ-Short than the comparison group (W = 82774, p < .001), with a comparable effect size of d = -0.47.

Age Matched Samples.

Age matching was done using the nearest method and resulted in the exclusion of n = 76 individuals with ASD. The resulting subgroups still differed significantly on age (ASD: 41.6 (13.5) years, Controls: 38.4 (15.0) years, t = 3.75, p < .001). Group differences in the SPQ-Short in this sample were still present (W = 119438, p < .001), with a comparable effect size (d = -0.46). In order to improve the matching, we performed a median-split on distance and repeated analyses on the groups with small distances. Those groups still differed significantly on age (t = -2.00, p = .046), however not the ASD group was younger (ASD: 51.5 (14.0) years, Controls: 52.9 (14.7) years). Group differences on the SPQ-Short were still present (W = 34381, p < .001), however the effect size was smaller (d = -0.26).
